# PFA toolbox: a MATLAB tool for Metabolic Flux Analysis

**DOI:** 10.1186/s12918-016-0284-1

**Published:** 2016-07-11

**Authors:** Yeimy Morales, Gabriel Bosque, Josep Vehí, Jesús Picó, Francisco Llaneras

**Affiliations:** MICElab, IIIA, Universitat de Girona, Campus Montilivi, P4, Girona, 17071 Spain; Institut Universitari d’Automàtica i Informàtica Industrial, Universitat Politècnica de València, Camino de Vera s/n, Edificio 5C, 46022 Valencia, Spain

**Keywords:** Metabolic Flux Analysis, Interval MFA, Possibilistic MFA, Constraint-based modelling

## Abstract

**Background:**

Metabolic Flux Analysis (MFA) is a methodology that has been successfully applied to estimate metabolic fluxes in living cells. However, traditional frameworks based on this approach have some limitations, particularly when measurements are scarce and imprecise. This is very common in industrial environments. The PFA Toolbox can be used to face those scenarios.

**Results:**

Here we present the PFA (Possibilistic Flux Analysis) Toolbox for MATLAB, which simplifies the use of Interval and Possibilistic Metabolic Flux Analysis. The main features of the PFA Toolbox are the following: (a) It provides reliable MFA estimations in scenarios where only a few fluxes can be measured or those available are imprecise. (b) It provides tools to easily plot the results as interval estimates or flux distributions. (c) It is composed of simple functions that MATLAB users can apply in flexible ways. (d) It includes a Graphical User Interface (GUI), which provides a visual representation of the measurements and their uncertainty. (e) It can use stoichiometric models in COBRA format. In addition, the PFA Toolbox includes a User’s Guide with a thorough description of its functions and several examples.

**Conclusions:**

The PFA Toolbox for MATLAB is a freely available Toolbox that is able to perform Interval and Possibilistic MFA estimations.

**Electronic supplementary material:**

The online version of this article (doi:10.1186/s12918-016-0284-1) contains supplementary material, which is available to authorized users.

## Background

The problem of estimating unknown metabolic fluxes in living cells has been tackled using several methodologies. MFA is one of the most extensively and successfully applied approaches to estimating fluxes [1]. Usually MFA refers to 13C-MFA which uses stable isotopically labeled substrates (e.g., 13C-labeled glucose) combined with stoichiometric balancing to estimate the metabolic fluxes in steady state systems [2, 3]. However, in this study we refer to non-13C-MFA methods. These methods mainly rely on measurements of external fluxes (uptake and production rates) to estimate the flux state of cells. Traditional MFA methods present some limitations when accounting for irreversible reactions [4], underdetermined problems [5], and lack of measurements [6]. To reduce these limitations we have developed Interval [7] and Possibilistic [8] MFA methods, which are well-suited methodologies for scenarios with limited available data. Their main benefits are the following [6–10]: (a) They can consider the irreversibility of the reactions and other inequality constraints. (b) They are able to represent the measured fluxes as intervals and even distributions to describe the uncertainty of the system. (c) They provide interval estimates, which are more reliable and more informative than pointwise solutions, particularly when multiple flux values are possible. (d) They are able to perform estimations in scenarios of high uncertainty or lack of measurements, being those estimates as reliable as possible. In addition, (e) Possibilistic MFA allows the detection and handling of inconsistencies between a model and a set of measurements. The PFA Toolbox provides all these features while preserving computational efficiency.

In the last years, several published works have used these methodologies to perform interval estimations of metabolic fluxes [9, 11–18] and consistency analysis with Possibilistic MFA [9, 17, 18]. Interval MFA was also implemented in FASIMU [16]. However, any intermediate user of MATLAB, Mathematica, R, etc. can easily implement Interval MFA. The easily implementation of Interval MFA has led to be used more often than Possibilistic MFA, which requires more mathematical development and additional linear optimizations. The PFA Toolbox presented here simplifies the use of both methods.

The PFA Toolbox provides a comprehensive set of MATLAB functions to easily and quickly apply Interval and Possibilistic MFA. The PFA Toolbox is completely free and open source; users are welcome to modify and adapt the toolbox code to build their own particular functions to fulfill specific requirements under the mild conditions described in the accompanying license. In the following subsections, we briefly describe the methods implemented in the toolbox: Interval MFA and Possibilistic MFA. A detailed description of both methods can be found in [6].

### Interval MFA

Interval MFA is a simple yet powerful extension of traditional MFA methods. It starts with a stoichiometric model or providing model-based constraints, denoted in the sequel as MOC, defined by a stoichiometric matrix **N** and a set of irreversibility constraints. These together define a space of feasible steady-state flux distributions [19, 20] (matrices and vector are denoted in bold):

1 $$ MOC=\left\{\begin{array}{c}\hfill N\cdot v=0\hfill \\ {}\hfill\ D\cdot v\ge 0\ \hfill \end{array}\right. $$

where, considering a system with n metabolites and r reactions, **N** ∈ **R**^{***nxr***}^ and **D** ∈ **R**^{*rxr*}^ is a diagonal matrix with **D**_**ii**_ = 1 if the flux is reversible (0 otherwise), and **v** ∈ **R**^{*r*}^ is the vector of metabolic fluxes. The values of **v** that are solution of (1) define a flux distribution.

Consider now a subset **v**_**m**_ ∈ **R**^***m***^ of measured fluxes in **v** with m typically much smaller than r. Following the interval approach, we represent each measured flux as an interval with inequalities:

2 $$ {\mathbf{v}}_{\boldsymbol{m}}^{\boldsymbol{m}}\le {\mathbf{v}}_{\boldsymbol{m}}\le {\mathbf{v}}_{\boldsymbol{m}}^{\boldsymbol{M}} $$

where **v**_***m***_^***m***^ and **v**_***m***_^***M***^ are vectors with the minimum and maximum possible values that the measured fluxes **v**_***m***_ can take due to measurement’s uncertainty.

Equations (1–2) describe a constraint-based model (CB) that defines the space of feasible fluxes. From this CB, the interval of feasible (possible) values for any flux v_i_ in the flux distribution **v** can be obtained solving two Linear Programming (LP) problems, as follows:

3 $$ \begin{array}{c}\hfill {\mathrm{v}}_{\mathrm{i}}^{\mathrm{m}}= \min {\mathrm{v}}_{\mathrm{i}}\kern0.5em s.t.\ v\ \in \left\{\begin{array}{c}\hfill MOC\hfill \\ {}\hfill {\mathbf{v}}_{\boldsymbol{m}}^{\boldsymbol{m}}\le {\mathbf{v}}_{\mathbf{m}}\le {\mathbf{v}}_{\boldsymbol{m}}^{\boldsymbol{M}}\hfill \end{array}\right.\hfill \\ {}\hfill {\mathrm{v}}_{\mathrm{i}}^{\mathrm{M}}= \max {\mathrm{v}}_{\mathrm{i}}\kern0.5em s.t.\ v\ \in \left\{\begin{array}{c}\hfill MOC\hfill \\ {}\hfill {\mathbf{v}}_{\boldsymbol{m}}^{\boldsymbol{m}}\le {\mathbf{v}}_{\mathbf{m}}\le {\mathbf{v}}_{\boldsymbol{m}}^{\boldsymbol{M}}\hfill \end{array}\right.\hfill \end{array} $$

This procedure provides an interval estimate for any flux of interest. These interval estimates are particularly useful in the two situations of having imprecise measurements and/or when few measures are available. Extra details about Interval MFA can be found in [6, 7, 10].

### Possibilistic MFA

Possibilistic MFA may be seen as a more flexible and powerful extension of Interval MFA. The methodology is based on two ideas: (a) Representing knowledge with constraints satisfied to a certain degree, thus transforming the feasibility of a potential solution into a gradual notion of “possibility” that accounts for uncertainty, and (b) using computationally efficient optimization-based methods, such as Linear Programming, to query for the “most possible” solutions. This methodology is able to face two different problems: (a) To evaluate the consistency between a model and a set of measurements, and (b) to obtain rich estimates of metabolic fluxes. Instead of pointwise estimates, it computes interval estimations for a desired degree of possibility and for entire possibility distributions.

Possibilistic MFA starts with a set of model-based constraints (MOC) defined in (1).

In this case, however, instead of using the simple inequalities (2), the measurements are incorporated in possibilistic terms by means of a set of constraints and two non-negative slack variables that represent the measurement’s uncertainty. These constraints, which we call measurement constraints (MEC), can be expressed as:

4 $$ MEC = \left\{\begin{array}{c}\hfill {\boldsymbol{w}}_{\boldsymbol{m}}={\mathbf{v}}_{\mathbf{m}}+{\boldsymbol{\varepsilon}}_1-{\boldsymbol{\mu}}_1+{\boldsymbol{\varepsilon}}_2-{\boldsymbol{\mu}}_2\hfill \\ {}\hfill {\boldsymbol{\varepsilon}}_1,{\boldsymbol{\mu}}_1\ge 0\hfill \\ {}\hfill 0\le {\boldsymbol{\varepsilon}}_2\le {\boldsymbol{\varepsilon}}_2^{\boldsymbol{m}\boldsymbol{ax}}\hfill \\ {}\hfill 0\le {\boldsymbol{\mu}}_2\le {\boldsymbol{\mu}}_2^{\boldsymbol{m}\boldsymbol{ax}}\hfill \end{array}\right. $$

where **v**_**m**_ is the vector of the actual values of the measured fluxes, and **w**_**m**_ is the vector of the measured values for them. Both differ due to errors and imprecisions. This uncertainty is represented by the slack variables ***ε***_***1***_**,*μ***_***1***_**,*ε***_***2***_ and ***μ***_***2***_. The bounds ***ε***_***2***_ and ***μ***_***2***_ define a band of fully possible values for **v**_**m**_ around the measured values **w**_**m**_. The components ***ε***_***1***_ and ***μ***_***1***_ are penalized in a cost index (5) to assign a decreasing possibility to larger errors. Each candidate solution of (1) and (4) can be denoted as δ = {**v, w**_**m**_**,*ε***_***1***_**,*μ***_***1***_**,*ε***_***2***_**,*μ***_***2***_}.

Now, we define a function, *π* (δ):∆ → [0,1] that assigns possibility *π* in [0, 1] to each solution, ranging from impossible to fully possible. A simple way to build this function is using a linear cost index *J* to penalize large deviations between the actual values of the fluxes and their measured ones:

5 $$ J=\boldsymbol{\alpha} \cdot {\boldsymbol{\varepsilon}}_1+\boldsymbol{\beta} \cdot {\boldsymbol{\mu}}_1 $$

The possibility of each solution is defined as:

6 $$ \pi \left(\delta \right)= \exp \left(-J\left(\delta \right)\right)\ \delta\ \epsilon\ MEC\ {\displaystyle \cap }MOC $$

Where **α** and **β** are row vectors of accuracy coefficients or weights that define each measurement’s a priori accuracy. These weights need to be defined by the user, e.g., if sensor error is «symmetric», **α** and **β** should be defined to be equal.

From this point, Possibilistic MFA calculates different estimates by solving LP problems. You can compute the set of flux values with maximum possibility (a pointwise estimation) or a more informative estimation with intervals or flux distributions.

### Pointwise estimations

The simplest outcome of a Possibilistic MFA problem is a pointwise estimate. It corresponds to the flux values with the maximum possibility (minimum cost), which are obtained by minimizing *J* and solving the LP problem:

7 $$ {J}_{\min }=\underset{\boldsymbol{\varepsilon}, \boldsymbol{\mu}, \boldsymbol{v}}{ \min }J=\boldsymbol{\alpha} \cdot {\boldsymbol{\varepsilon}}_1+\boldsymbol{\beta} \cdot {\boldsymbol{\mu}}_1\ s.t\ \left\{MOC{\displaystyle \cap }MEC\right.\Big\} $$

The solution flux vector **v**, that we call **v**_**mp,**_ contains the most possible values that are consistent with both the model and the measurements.

This pointwise estimation may be unreliable when multiple solutions are reasonably possible. In these instances, distributions and interval estimates can be computed instead.

### Interval estimates

The interval estimate [v_γ_^m^, v_γ_^M^ ] for a flux v, with a conditional possibility higher than γ, can be computed solving two extra LP’s:

8 $$ {\mathbf{v}}_{\gamma}^m={ \min}_{\boldsymbol{\upvarepsilon}, \boldsymbol{\upmu}, \mathbf{v}}\mathrm{v}\ s.t\left\{\begin{array}{c}\hfill MOC{\displaystyle \cap }MEC\hfill \\ {}\hfill J-{J}_{\min }<- \ln \gamma \hfill \end{array}\right. $$

The upper bound is defined by replacing minimum for maximum.

### Distributions as estimates

The complete possibility distribution of a flux can also be obtained for marginal and conditional possibilities. Marginal possibilities provide the degree of possibility of each value for a given flux. Conditional distributions are equivalent to normalizing the marginal possibility distribution to a maximum equal to one.

Possibilistic MFA was casted as a linear optimization problem, for which widely known and efficient tools exist. This great computational performance makes the methodology suitable —in principle— for large-scale metabolic networks.

More information about the methods and a deeper discussion about the strengths and limitations of each approach can be found in our previous works [6–8, 10] and in the toolbox User’s Guide (http://kikollan.github.io/PFA-Toolbox/).

## Implementation

The PFA Toolbox has been developed to run in MATLAB. Its core is a set of MATLAB functions that solve each step in a typical MFA problem. The code for all functions is provided with the toolbox. The PFA Toolbox also includes a Graphical User Interface (GUI) to represent the measurements in possibilistic terms. The GUI runs within MATLAB.

The toolbox requires solving LP problems, and those are solved with a flexible and efficient external optimizer, YALMIP [21]. We provide a copy of YALMIP within the PFA Toolbox, but further information about it can be found at the YALMIP website [22]. YALMIP can use different LP solvers, and so does the PFA Toolbox. Three LP solvers were tested: IBM ILOG CPLEX by IBM [23], GLPK [24], and Linprog, the LP solver included in MATLAB. However, we do not recommend the use of Linprog, which proved unreliable, especially for larger MFA problems. Instead, CPLEX or GLPK showed excellent performance. CPLEX has a 90-day free evaluation version, and can be used free for research and academic purposes. GLPK is freely available.

## Results and discussion

In this section, we show how to use the PFA Toolbox for MATLAB. A list of the functions provided by the toolbox is shown in Table 1. These functions simplify the process of (1) defining the MFA problem, (2) computing different types of estimates (pointwise, interval or distributions) and (3) plotting the results. There is also a function to plot the measurements defined in possibilistic terms, and a GUI to define those measurements. Advanced users can modify and extend each function.

**Table 1:** List of functions in the PFA Toolbox

Initialization	
initPFAtoolbox	It starts the PFA Toolbox
1: MFA problem formulation	
define_MOC	It defines the model-based constraints
define_PossMeasurements	It represents the measured fluxes
define_MEC	It defines the measured-based constraints
2: Computing estimations	
solve_maxPoss	It calculates the most possible set of flux values
solve_maxPossIntervals	It calculates the interval of most possible flux values
solve_PossInterval	It calculates the interval of flux values with the desired possibility
3: Plotting the estimations	
plot_PossMeasurements	It plots measurements in possibilistic terms
plot_distribution	It plots the distribution of a given flux
plot_intervals	It plots interval estimates of a given flux
4: Other	
Solve_possintervalYMP	Advanced function; read its help.
solve_Interval	It solves an Interval MFA problem

The main features of the PFA Toolbox are the following:

- » It gives reliable MFA estimations even in uncertain or underdetermined scenarios (those where only a few fluxes can be measured).
- » It provides MFA estimations accounting for measurement’s imprecision.
- » It provides functions to plot interval estimates and distributions.
- » It is composed of simple, free and open functions.

**Fig. 1:**
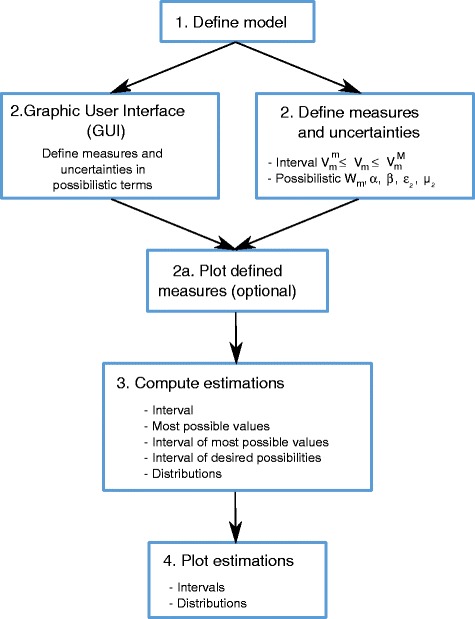
Protocol to use the PFA Toolbox. A step by step to use the PFA Toolbox. Protocol is the same to solve the MFA problems with Interval and possibilistic MFA. Possibilistic has two additional steps, which are optional, a Graphical User Interface (GUI) to represent graphically the measures in possibilistic terms and a function to check if the measures and their uncertainties are well-defined

 A step-by-step protocol to apply Interval or Possibilistic MFA is presented in Fig. 1.

In addition, the toolbox is developed to use stoichiometric models with the format of the COBRA Toolbox (Constraint-Based Reconstruction and Analysis). This format is widely used due to the popularity of COBRA. As an alternative, the user can simply define a model by providing a stoichiometric matrix.

The main features of the toolbox are shown in the next three examples. Additional examples and a thorough description of all functionalities of the toolbox are provided in the User’s Guide. The details about the mathematical methods implemented in the toolbox can be found in [7, 8, 10], and in [6].

### Example of flux estimation under data scarcity

**Fig. 2:**
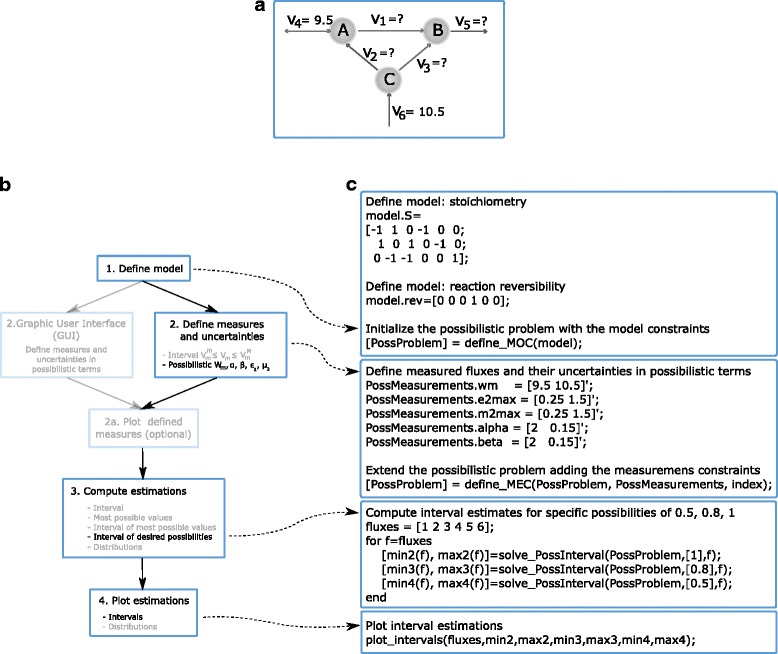
PFA Toolbox methodology to solve example of flux estimation under data scarcity. **a** Upper panel present a simple metabolic network. Metabolites are in capital letters, each v_j_ represent a flux and the double arrows indicate a reversible reaction. **b** The step-by-step procedure follow to solve the MFA problem where only two measures are known. **c** Right panel shows the MATLAB code used to perform the computations

We use a toy metabolic network to illustrate how to use the PFA Toolbox in scenarios of data scarcity. The first step is to formulate the problem. Consider the metabolic network shown in Fig. 2a. The network has six fluxes and three balanced metabolites. One of the fluxes is reversible. Additionally, the fluxes v_4_ and v_6_ have been measured, with values w_4_ = 9.5 mmol/h, and w_6_ = 10.5 mmol/h.

The MFA problem consists in the estimation of all six fluxes. Notice, however, that traditional MFA cannot be performed because the problem is undetermined: any pointwise estimate will be only a particular solution of a group of possible ones [5]. The methods in the PFA Toolbox tackle this situation and provide reliable and informative estimates.

In this case, we choose to apply Possibilistic MFA to estimate the fluxes. The first step to solve the problem is to define the model-based constraints (MOC). Stoichiometric model can be directly defined in the code or be provided in COBRA format.

The next step is the addition of measurements and their uncertainties (in this example, we assume that the measurement w_4_ is very accurate, but w_6_ is not. In agreement with the problem formulation, we assign values to the slack variables μ_2_ and ε_2_, and the weights α and β (details about this process can be found in the User’s Guide).

Once the MOC and MEC constraints have been defined, the third step is to obtain the estimates. Possibilistic MFA methodology calculates three types of estimations. In this case, we compute three interval estimates for each flux, for conditional possibilities of 0.5, 0.8 and 1.

**Fig. 3:**
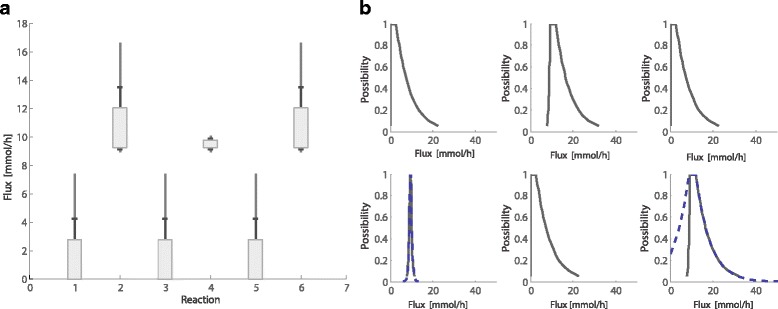
Flux estimation. Estimations for every flux were obtained with the PFA Toolbox. **a** Three interval estimates are given, for maximum conditional possibility (box), possibility of 0.8 (black line), and 0.5 (gray line). **b** Possibility distributions are depicted with solid lines and dashed lines represent measured values

Finally, we plot the interval estimates using the function *plot_intervals*. The metabolic network and the main features of the algorithm to solve the problem with the PFA Toolbox are shown in Fig. 2. Figure 3a shows the interval estimations for each dataset. Notice that even if only two measurements are available, the estimation is reliable.

This same procedure can be applied to obtain other types of estimates, such as the complete possibility distribution for a flux. Those computations can be performed using the function *solve_PossInterval*. The obtained distributions are for conditional possibilities (see [8] for a detailed explanation of the notion of conditional possibility). These possibilistic distributions can be plotted with the fuction *plot_distribution*. As an example, Fig. 3b shows the distribution estimation for all the six fluxes. The results show, for instance, that the most possible value for v_1_ is 2.75 mmol/h (π = 1), that v_1_ being equal to 6.1 mmol/h is a less possible situation (π = 0.6), and that a v_1_ being larger than 18 mmol/h is very unlikely (π < 0.1).

The model and the code for all the computations are provided as (Additional file 1a).

Note: to apply Interval MFA a similar protocol can be followed. The main difference is that the measures will be represented as intervals instead of being represented in possibilistic terms.

### Example of flux estimation: biomass growth of *Pichia pastoris*

In this example, we estimate the growth of several chemostat cultures of *P. pastoris*. For each chemostat only a few extracellular fluxes are measured (mainly substrates uptakes and secretion rates) and the aim is to estimate the cellular growth.

The constraint-based model for *P. pastoris* used is presented in [18] (see Additional file 2). It is a relatively small representation including only the main catabolic pathways considering the uptake of the usual carbon sources: methanol, glucose and glycerol. The stoichiometric model contains 37 metabolites and 48 reactions, with reversibility accounted for. The stoichiometric matrix and all the measurements can be found in the (Additional file 3) [31-35].

**Fig. 4:**
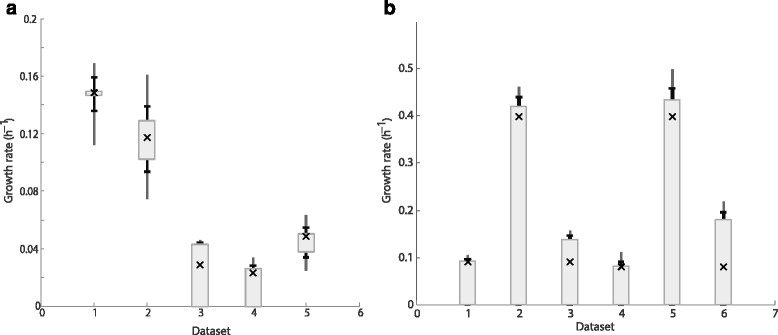
Growth estimations with possibilistic MFA for *P. pastoris* and *E. coli*. **a** Example with six *P. pastoris* experiments. **b** Example with *E. coli* experiments. In both cases, three interval estimates are represented, for conditional possibilities equal to 0.99 (box), 0.5 (bar) and 0.1 (line). The crosses represent the actual experimental values

We select to apply Possibilistic MFA to perform the estimation. As before, we start by defining the MOC and MEC constraints. In this example, we assign the same uncertainty to all the measurements: a deviation of 5 % around the measured value is assumed to be fully possible, while a deviation larger than 20 % is assumed to be an event of low possibility (π = 0.1). The next step is to estimate the growth for each experiment. We compute three interval estimates for conditional possibilities of 0.99, 0.5 and 0.1. Finally, we plot the interval estimates, results are shown in Fig. 4a.

The estimations show good agreement with the experimental growth rates (as expected, since this model and the data have been tested previously). Notice that the interval estimates not only predict the growth rates but also provide an indication of the estimation reliability. The complete code for all computations can be found in the (Additional file 1b).

### Example of flux estimation: growth of *Escherichia coli*

Here we use a well-known model of *E. coli*, taken from [25] and illustrated in the (Additional file 4). It is a relatively compact model containing 72 metabolites and 95 reactions. We consider six chemostat experiments of *E. coli* growing in glucose [26]. The datasets contain information only for a handful of extracellular measurements (growth rate, substrate uptake, oxygen uptake, CO_2_ production and acetate and pyruvate secretion). The model and the measurements can be found in the (Additional file 5).

Possibilistic MFA is applied again to estimate the growth rate for all six scenarios. The problem is similar to the previous one, and we assume the same uncertainty for each measurement. However, we now consider a larger model for a different and widely used organism. The computation procedure is analogous to the one previously described. The complete code for all computations can be found in the (Additional file 1c).

The flux estimates computed with the toolbox are compatible with the actual growth rate in all scenarios (Fig. 4b). Notice, however, that the estimates are wider than in the first example (no-growth is possible in all of them, but the maximum possible growth is near the actual one). The model is larger and the available measurements are not enough to determine completely the flux state of cells. This illustrates one limitation of Interval and Possibilistic MFA: the estimates are only as precise as the uncertainty and the available measurements allow.

### Example of consistency analysis with *P. pastoris*

The last example illustrates how the PFA Toolbox can be used for another purpose: to evaluate the degree of consistency between a given model and a set of experimental measurements. Consider the data of six chemostat experiments with *P. pastoris* taken from the literature (Table 2). We test how consistent the data for each experiment are against the model of *P. pastoris* described previously. We assume that the model is reliable and therefore it can be used to evaluate the validity of each dataset. Notice that this is a strong assumption, valid here for the purpose of this example. It is indeed possible to perform the exact opposite analysis: to obtain several experimental datasets and use them to assess the quality of a metabolic model. We use Possibilistic MFA to validate the model of *P. pastoris* [9, 18]. The objective of the analysis performed here is to detect if there are (larger than expected) errors in the measurements.

**Table 2:** Experimental data for six chemostat experiments with *Pichia pastoris* and an analysis of its consistency against a model

Reference	μ	Q_Glu_	Q_Glyc_	Q_Met_	Q_Pyr_	Q_Cit_	Q_EtOH_	OUR	CPR	π_mp_^b^
	Cmmol^a^/g/h	mmol/g/h	mmol/g/h	mmol/g/h	mmol/g/h	mmol/g/h	mmol/g/h	mmol/g/h	mmol/g/h	
[31]	6.17	0.00	2.75	0.00	0.00	0.00	0.00	3.62	2.35	0.16
[32]	3.27	0.81	0.00	1.09	0.00	0.00	0.00	4.02	2.68	1.00
[33]	2.38	0.00	1.21	0.00	N.A.	N.A.	N.A.	1.65	1.22	1.00
[33]	4.89	0.00	2.40	0.00	N.A.	N.A	N.A.	3.12	2.29	1.00
[34]	1.40	0.00	0.00	2.55	N.A.	N.A.	N.A.	2.16	1.15	1.00
[34]	0.94	0.00	0.00	1.87	N.A.	N.A.	N.A.	1.67	0.93	1.00

^a^Cmmol = Carbon mmol ^b^Dimensionless value of the possibility of the most possible flux distribution

We start as in previous examples by defining MOC and MEC constraints. The next step is to compute the estimation. In this example, we compute the most possible solution for each experiment with the *solve_maxPoss* function. This provides the maximum possibility flux vector and the associated degree of possibility (π_mp_) between [0, 1] of the most possible solution. This value provides an indication of the agreement between the model-based constraints (MOC) and the measurements constraints (MEC).

A possibility equal to one is interpreted as a complete consistency; a lower value implies that there are errors in one (or more) of the measurements or in the model. The complete MATLAB code for this computation can be found in (Additional file 1b).

The results presented in Table 2 show that all datasets except one are highly consistent with the model. The dataset 1 has a low degree of possibility (lower 0.2). This suggests that one or more of the measured fluxes in that experiment is unreliable and may contain errors.

All the computations of these four examples were performed with the PFA Toolbox. The computations take approximately 13 s in a 64-bit Windows PC (Intel Core™ i5 2.5 GHz processor), using MATLAB R2012a with IBM ILOG CPLEX Optimizer as the solver for Linear Programming problems.

### Notes on computational efficiency and large networks

The methods used by the PFA Toolbox, Possibilistic MFA and interval MFA, have been cast as linear optimization problems, and thus they can be solved with computational efficiency. This makes these methodologies suitable for large-scale metabolic networks. For instance, when tested on a genome-scale *E. coli* model (iJO1366) that contains 2583 reactions [27], the PFA Toolbox is able to get estimates for all 2507 fluxes with three degrees of possibility (i.e., solving 3x2507 LP problems). Computing those estimates required 120 min in an AMD A10–5800 K with Radeon HD graphic (3.80 GHz) PC and 8 GB of RAM with GLPK optimizer. This suggests that the PFA Toolbox may be able to solve MFA flux estimations of large models with good results and reasonable computational cost.

There is, however, a limitation regarding MFA-wise methods when estimating fluxes in large networks: there may be too many flux vectors compatible with the (few) available measurements [28]. Unlike traditional methods, those proposed here may still be of use in this situation. Possibilistic MFA and Interval MFA capture all the equally possible flux states (or “similarly” possible) by means of possibilistic distributions or intervals. If there is a wide range of candidates, however, the estimation may be only slightly informative. If this is the case, one could decide to incorporate a rational assumption, as done in FBA methods [29, 30].

## Conclusions

We have presented the PFA Toolbox for MATLAB. This toolbox provides a set of MATLAB functions to apply Interval MFA and Possibilistic MFA in a simple and flexible way. The PFA Toolbox is completely free and open source, and can be modified by its users. The toolbox implements MFA-wise methods to perform metabolic flux estimations that are particularly well suited to deal with scenarios of high uncertainty and scarce measurements, which are common in industry.

## Availability and requirements

**Project name:** PFA Toolbox version 1.0.0.

**Project home page**: http://kikollan.github.io/PFA-Toolbox/

**Operating systems:** platform independent.

**Programming language:** MATLAB

**Other requirements: −**

**License:** Own license.

**Any restriction to use by non-academics:** none.

## Abbreviations

CB, constraint-based model; COBRA, Constraint-Based Reconstruction and Analysis; FASIMU, Flux-balance Analysis based Simulations; GLPK, GNU Linear Programming kit; GUI, Graphical User Interface; IBM ILOG CPLEX, High-performance mathematical programming solver for linear programming; LP, Linear Programming; MEC, Measurement constraints; MFA, Metabolic Flux Analysis; MOC, model-based constraints; PFA, Possibilistic Flux Analysis; YALMIP, Modelling language for advanced modeling and solution of optimization problems

## Additional files

Additional file 1: Code for the examples A.rar file with the MATLAB files code to perform the examples described below with Example of flux estimation under data scarcity (a), *P. pastoris* (b) and *E. coli* (c). (RAR 5 kb) Additional file 2: Metabolic network of *P. pastoris*. Metabolic network for the *Pichia pastoris* model. For the sake of clarity, the reactions representing biomass growth and ATP balance have not been included in the scheme. (PDF 1082 kb) Additional file 3: Stoichiometric matrix and experimental data for *Pichia pastoris.* A Microsoft Excel spreadsheet file with i) the list of reactions and metabolites, ii) the stoichiometric matrix of *P. pastoris* and iii) the experimental datasets taken from the literature. This includes measurements of biomass, substrates uptakes (glycerol, glucose, and methanol), Oxygen Uptake Rate (OUR), CO2 production (CPR), and formation of byproducts (ethanol, citrate, and pyruvate). (XLSX 48 kb) Additional file 4: Metabolic network of *Escherichia Coli.* Metabolic network for the *Escherichia coli* model. (PDF 86 kb) Additional file 5: Stoichiometric matrix and experimental data for Escherichia coli. A Microsoft Excel spreadsheet file with i) the stoichiometric matrix of *E. coli* and ii) the experimental datasets taken from the literature. This includes measurements of biomass, glycerol, OUR, CPR and pyruvate. (XLSX 62 kb)
