# Supplementary figures and images for: PFA toolbox: a MATLAB tool for Metabolic Flux Analysis
Source: BMC Syst Biol. 2016 Jul 11;10:46. doi: 10.1186/s12918-016-0284-1 (PMC4940746; doi:10.1186/s12918-016-0284-1)

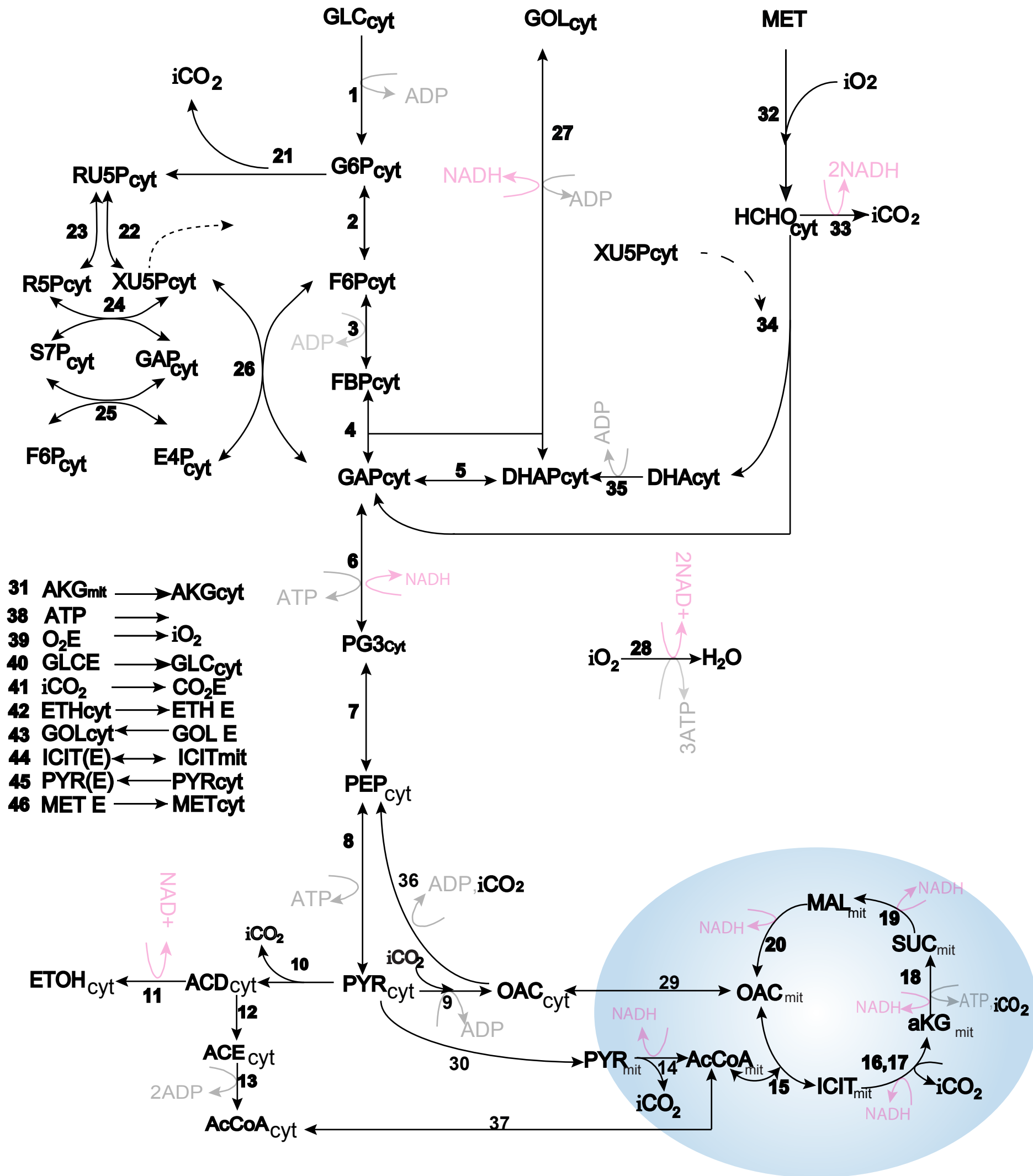

Supplement: Additional file 2: — Metabolic network of P. pastoris. Metabolic network for the Pichia pastoris model. For the sake of clarity, the reactions representing biomass growth and ATP balance have not been included in the scheme. (PDF 1082 kb) [file 12918_2016_284_MOESM2_ESM.pdf]

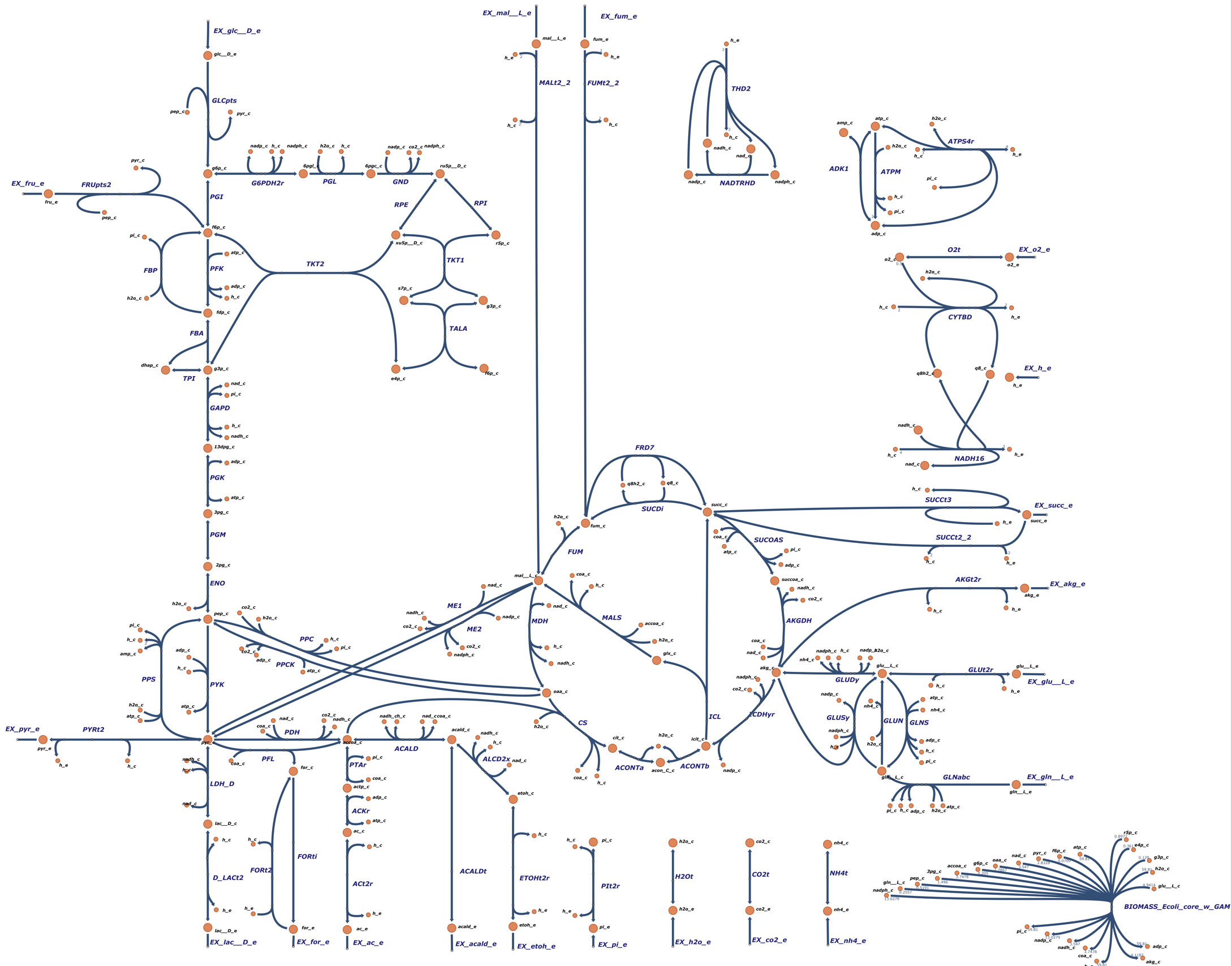

Supplement: Additional file 4: — Metabolic network of Escherichia Coli. Metabolic network for the Escherichia coli model. (PDF 86 kb) [file 12918_2016_284_MOESM4_ESM.pdf]
